# Measuring Peripheral Chemoreflex Hypersensitivity in Heart Failure

**DOI:** 10.3389/fphys.2020.595486

**Published:** 2020-12-29

**Authors:** Daniel A. Keir, James Duffin, John S. Floras

**Affiliations:** ^1^ University Health Network and Mount Sinai Hospital Division of Cardiology and Department of Medicine, University of Toronto, Toronto General Research Institute, Toronto, ON, Canada; ^2^ School of Kinesiology, The University of Western Ontario, London, ON, Canada; ^3^ Department of Anesthesia and Pain Management, University of Toronto, Toronto, ON, Canada; ^4^ Department of Physiology, University of Toronto, Toronto, ON, Canada; ^5^ Thornhill Research Inc., Toronto, ON, Canada

**Keywords:** carotid body, chemoreceptors, hypercapnia, hypoxia, sympathetic nervous system, ventilation

## Abstract

Heart failure with reduced ejection fraction (HFrEF) induces chronic sympathetic activation. This disturbance is a consequence of both compensatory reflex disinhibition in response to lower cardiac output and patient-specific activation of one or more excitatory stimuli. The result is the net adrenergic output that exceeds homeostatic need, which compromises cardiac, renal, and vascular function and foreshortens lifespan. One such sympatho-excitatory mechanism, evident in ~40–45% of those with HFrEF, is the augmentation of carotid (peripheral) chemoreflex ventilatory and sympathetic responsiveness to reductions in arterial oxygen tension and acidosis. Recognition of the contribution of increased chemoreflex gain to the pathophysiology of HFrEF and to patients’ prognosis has focused attention on targeting the carotid body to attenuate sympathetic drive, alleviate heart failure symptoms, and prolong life. The current challenge is to identify those patients most likely to benefit from such interventions. Two assumptions underlying contemporary test protocols are that the ventilatory response to acute hypoxic exposure quantifies accurately peripheral chemoreflex sensitivity and that the unmeasured sympathetic response mirrors the determined ventilatory response. This Perspective questions both assumptions, illustrates the limitations of conventional transient hypoxic tests for assessing peripheral chemoreflex sensitivity and demonstrates how a modified rebreathing test capable of comprehensively quantifying both the ventilatory and sympathoneural efferent responses to peripheral chemoreflex perturbation, including their sensitivities and recruitment thresholds, can better identify individuals most likely to benefit from carotid body intervention.

## Introduction

The carotid bodies, located at the bifurcation of the carotid artery, contain chemosensitive cells (type I glomus cells) that respond to increased hydrogen ion concentration ([H^+^]) and decreased oxygen pressure (PO_2_) within their intracellular environment by releasing neuro-active agents to stimulate the carotid sinus nerve (Ortega-Sáenz and López-Barneo, 2020). The resulting increase in afferent input to the medulla elicits reflexive changes in both ventilation, to restore arterial PO_2_ (PaO_2_) and pH *via* a reduction in arterial carbon dioxide pressure (PaCO_2_; Prabhakar, 2013; Guyenet, 2014), and sympathetic discharge, to counter the direct vasodilatory effects of hypercapnia and hypoxia (Ainslie and Duffin, 2009; Keir et al., 2019).

The heightened sympathetic drive characteristic of heart failure with reduced ejection fraction (HFrEF) reflects integration of compensatory baroreceptor reflex disinhibition, in response to a fall in cardiac output, and patient-specific activation of one or more excitatory stimuli arising from, for example, afferents in the atria, skeletal muscle, and kidneys (Floras and Ponikowski, 2015). The result is a net adrenergic response that exceeds homeostatic need; compromises cardiac, renal, and vascular function; and foreshortens lifespan (Floras and Ponikowski, 2015). Augmented carotid (peripheral) chemoreceptor sensitivity is one such maladaptive mechanism HFrEF (Floras and Ponikowski, 2015; van Bilsen et al., 2017). Consequently, there is an increasing interest in interventions that target the peripheral chemoreflex with the aim of moderating efferent sympathetic traffic, alleviating heart failure symptoms and prolonging life expectancy (Narkiewicz et al., 1999a; Niewinski et al., 2013; Paton et al., 2013; Schultz et al., 2013; Del Rio et al., 2015; Niewinski, 2017; Toledo et al., 2017; van Bilsen et al., 2017).

In a substantial proportion of patients with chronic HFrEF, either increased tonic chemoreceptor activity or augmented chemoreceptor reflex responsiveness to changes in PaO_2_ or PaCO_2_ (the latter stimulus applied as a pragmatic surrogate for [H^+^]), or both, contribute to upward resetting of the resting efferent sympathetic outflow (Di Vanna et al., 2007; Hering et al., 2007; Floras, 2009; Despas et al., 2012). For example, increased tonic activity, evident as an acute reduction in muscle sympathetic nerve activity (MSNA) when the carotid body of afferent nerve activity is attenuated by inhalation of 100% O_2_, may be present in up to 40% of patients with HFrEF (van de Borne et al., 1996; Ponikowski et al., 2001; Andreas et al., 2003; Hering et al., 2007; Franchitto et al., 2010; Despas et al., 2012).

The subject of this perspective is not such tonic activation, but rather peripheral reflex gain or sensitivity, calculated conventionally as the ventilatory response to an acute hypoxic or hypercapnic stimulus. This has received much more attention in the HFrEF literature because of its high prevalence; the rapid fluctuations in PaCO_2_ and PaO_2_ that can occur consequent to changes in activity, behavior, and emotion; and the reported association of the hypoxic ventilatory response (HVR) with prognosis. With respect to the latter, several groups have estimated that, relative to healthy subjects, peripheral chemoreflex sensitivity is augmented in up to 45% of patients with HFrEF (Narkiewicz et al., 1999b; Ponikowski et al., 2001; Giannoni et al., 2008). Such augmented hypoxic ventilatory responsiveness associates, independently, with HFrEF severity (Giannoni et al., 2008), the magnitude of MSNA (Ponikowski et al., 2001; Di Vanna et al., 2007), the presence of disordered breathing (Ponikowski et al., 1999; Solin et al., 2000; Marcus et al., 2014a,b), blunted baroreflex sensitivity (Ponikowski et al., 1997), and, in adjusted models, with foreshortened life expectancy (Ponikowski et al., 1999, 2001; Schultz and Li, 2007; Giannoni et al., 2009).

These findings have stimulated studies of the acute or chronic consequences of blunting chemoreceptor activity or carotid body resection. In a canine HFrEF model, carotid body chemoreceptor inhibition by infused dopamine increased resting hind-limb vascular conductance immediately (preceding the systemic effects of dopamine) and more so than in healthy dogs (Stickland et al., 2007). Carotid body denervation reduced resting and hypoxia-induced renal sympathetic nerve activity, disordered breathing patterns, and arrhythmia incidence in HFrEF rabbits (Marcus et al., 2014b) and rats (Del Rio et al., 2013) and improved their survival (Del Rio et al., 2013). Narkiewicz et al. (2016) reported reductions in ambulatory blood pressure and resting MSNA following unilateral carotid body resection in 8 of 15 patients with drug-resistant hypertension. Interestingly, those with a positive outcome (i.e., responders) had a greater preoperative ventilatory responsiveness to hypoxia. In a first-in-man HFrEF study of unilateral (*n* = 4) or bilateral (*n* = 6) carotid body resection, ventilatory responsiveness to decreased inspired O_2_ was 70% lower, 1 month after surgery, and resting MSNA fell by 9% (Niewinski et al., 2017). Such findings promoted the concept of carotid body excision as a therapeutic option to redress autonomic disequilibrium or the occurrences of central apnea.

Since eliminating oxygen sensing is not without risk (Smit et al., 2002; Timmers et al., 2003; Narkiewicz et al., 2016), the challenge is to identify those individuals most likely to benefit. Determining the HVR has been proposed for this purpose (Paton et al., 2013; Narkiewicz et al., 2016; Niewinski, 2017). Two assumptions underlying this strategy and the contemporary test protocols employed are: (1) that the ventilatory response to acute hypoxic exposure quantifies accurately peripheral chemoreflex sensitivity and (2) that the unmeasured sympathetic response is congruent with the determined ventilatory response. This perspective will question both assumptions, illustrate with two HFrEF patients as examples the limitations of conventional transient hypoxic tests for assessing peripheral chemoreflex sensitivity, and demonstrate how a modified rebreathing test (as detailed in the accompanying Supplementary Material) capable of comprehensively quantifying both the ventilatory and sympathoneural efferent responses to peripheral chemoreflex perturbation, including their sensitivities and recruitment thresholds, can better identify individuals with heart failure most likely to benefit from carotid body intervention.

## How has Peripheral Chemoreflex Sensitivity Been Assessed in HFrEF?

Conventionally, in humans, peripheral chemoreceptor-specific responsiveness has been quantified by recording breath-by-breath ventilation (V̇_E_) and arterial O_2_ saturation (S_a_O_2_) during a transient hypoxic challenge. Popular in the HFrEF literature are protocols that lower O_2_ intermittently by nitrogen (N_2_) gas inhalation or continuously by rebreathing (Chua et al., 1996, 1997; Ponikowski et al., 1997, 1999; Niewinski et al., 2014). In the intermittent hypoxia test, a variety of exposure durations (~2–8 breaths of N_2_) are used to achieve a range of O_2_ saturations (70–100% S_a_O_2_). The peak in V̇_E_ subsequent to each exposure duration is plotted against the nadir in S_a_O_2_. The slope of the linear regression applied to the resultant V̇_E_–S_a_O_2_ relationship defines the HVR in L∙min^−1^∙%S_a_O_2_^−1^ (Edelman et al., 1973). The same HVR relationship may also be constructed continuously by having participants rebreathe from a system that facilitates a smooth fall in O_2_ and simultaneous CO_2_ removal to prevent its accumulation (with the aim of minimizing O_2_ vs. CO_2_ specific responses; Rebuck and Campbell, 1974).

Applying either method, investigators report that relative to healthy age-matched controls, whose HVR is approximately ~0.35 L∙min^−1^∙%S_a_O_2_^−1^, the mean HVR of cohorts with HFrEF is on average more than two standard deviations greater (Chua et al., 1996, 1997), and consistently circa 0.75 L∙min^−1^∙%S_a_O_2_^−1^ (Ponikowski et al., 1999, 2001). Notably, compared to HFrEF patients with HVR below this value, patients with HVR values in excess of 0.77 L∙min^−1^∙%S_a_O_2_^−1^ (Giannoni et al., 2009) and 0.72 L∙min^−1^∙%S_a_O_2_^−1^ (Ponikowski et al., 2001) were found to have survival reduced by 12 and 36% over 4 and 3 years, respectively. Consequently, in patients with HFrEF, an “exaggerated” HVR response is now defined as a value ≥0.75 L∙min^−1^∙%S_a_O_2_^−1^ (Giannoni et al., 2008, 2009; Niewinski, 2017). This threshold has been proposed for the selection of patients for carotid body interventions (Narkiewicz et al., 2016; Niewinski, 2017).

Importantly, because such transient hypoxic protocols fail to control for concurrent changes in PCO_2_, which will independently alter [H^+^] at the peripheral and central chemoreceptors (Nielsen and Smith, 1952; Hornbein and Roos, 1963; Cunningham et al., 1986; Guyenet, 2014), they often misrepresent peripheral chemoreflex sensitivity. For example, with intermittent delivery, the hypocapnia that accompanies longer N_2_ exposures will blunt the ventilatory response to hypoxia, leading to a lower calculated HVR. With the continuous delivery method, attempts have been made to maintain isocapnic conditions by removing excess CO_2_ during rebreathing. However, the choice of isocapnic PCO_2_ often is based on resting PCO_2_, which may reside above or below the PCO_2_ threshold at which the peripheral chemoreceptors initiate a ventilatory response to hypoxia (i.e., the ventilatory recruitment threshold, VRT; Duffin, 2007, 2011). Consequently, variation in the isocapnic PaCO_2_ at which patients are tested will alter HVR obscuring differences in peripheral reflex sensitivity between populations. To demonstrate these problems, in the next section, we report a single patient-participant experiment performed in our laboratory designed specifically to highlight the consequences of unstandardized PCO_2_ control when quantifying HVR.

## An Example of Peripheral Chemoreflex Sensitivity Assessment in HFrEF Using the Transient Hypoxic Test

A 26-year-old male with dilated cardiomyopathy (NYHA class II, LVEF = 20%; BMI = 30 kg∙m^−2^) and on optimal guideline recommended heart failure therapy, underwent four transient hypoxic tests under a poikilocapnic condition, and at three isocapnic PCO_2_ tensions. He was seated and breathed through a facemask connected in series to a low dead space, low air resistance pulmonary filter, and a bidirectional volume turbine (UVM, VacuMed, Ventura, CA, USA). The volume turbine measured expired volumes and was directly attached on its distal end to a sequential gas delivery circuit (Duffin, 2011).

Respired air was continuously sampled at the mouth and analyzed for the fractional concentrations of O_2_ and CO_2_ (17500B, VacuMed, Ventura, CA, USA). Respiratory volumes and fractional gas concentrations were recorded at a frequency of 50 Hz *via* a 16-bit analog-to-digital converter (National Instruments Inc., Austin, TX, USA) and then transferred to a computer. Custom software aligned the gas concentrations and volume signals and executed a peak-detection program to determine the end-tidal partial pressures of O_2_ (P_ET_O_2_) and CO_2_ (P_ET_CO_2_), tidal volumes, breathing frequencies and V̇_E_ on a breath-by-breath basis. Oxygen saturation (S_a_O_2_) was monitored at the ear using a pulse oximeter (Nonin 7500, Plymouth, MN, USA).

The sequential gas delivery circuit comprised a non-rebreathing valve, an expiratory gas reservoir, and an inspiratory gas reservoir supplied by a flow-controlled gas blender. A one-way crossover valve between the expiratory gas reservoir and the inspiratory limb permitted rebreathing of previously expired gas at the end of inspiration when ventilation exceeded the flow of fresh gas delivered into the circuit. In this way, the volume and composition of gas available for gas exchange was manipulated to allow precise control of alveolar ventilation and arterial blood gases independent of V̇_E_. To measure isocapnic HVR, the flow of fresh gas was fixed (which sets isocapnic P_ET_CO_2_), and the fractional composition of O_2_ was lowered progressively over the course of 90 s to achieve a decrement in S_a_O_2_ from 95 to 80%.

The HVR was determined under three isocapnic conditions: (1) at resting P_ET_CO_2_ (39 mmHg); (2) at +3 mmHg above resting P_ET_CO_2_ (42 mmHg); and (3) at +6 mmHg above resting P_ET_CO_2_ (45 mmHg). In a fourth test, HVR was measured without P_ET_CO_2_ control (poikilocapnia). As increasing P_ET_CO_2_ above resting levels will also increase central PCO_2_, a 4-min baseline period of isocapnic normoxia preceded each HVR test to ensure that ventilatory responses to PCO_2_ by the central chemoreceptors were complete before the induction of hypoxia. Further, to ensure that ventilatory drive from central chemoreceptors remained constant throughout hypoxic exposure, the HVR test was limited to 90 s to prevent hypoxia-mediated increases in cerebral blood flow from lowering central PCO_2_ (Duffin, 2007; Ainslie and Duffin, 2009).

**Figure 1:**
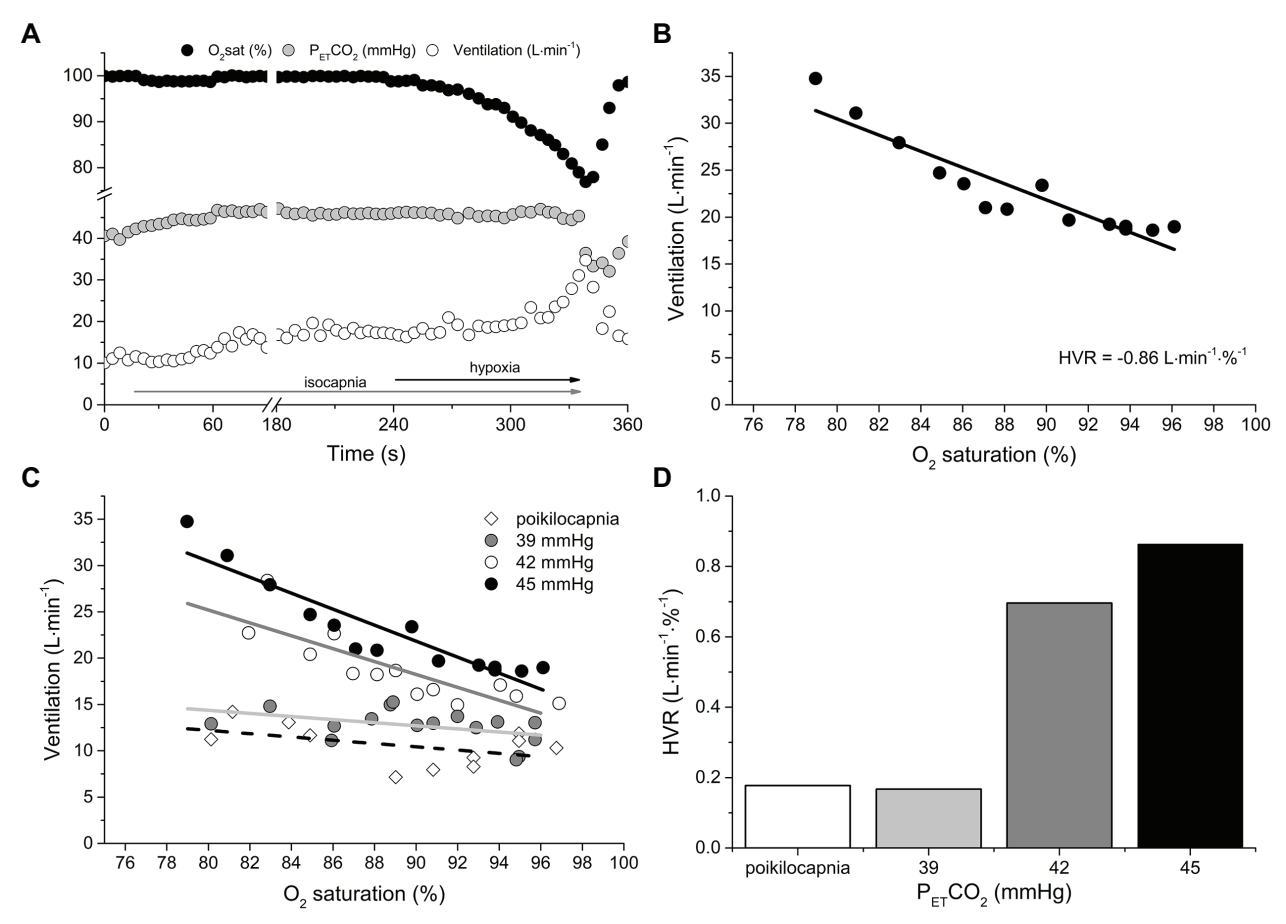
An example of the measurement of peripheral chemoreflex sensitivity using the continuous transient hypoxic method in an isocapnic condition (A). The rise in ventilation (V̇_E_) is plotted against the fall in oxygen saturation to build a regression function (B). The slope of this function, or hypoxic ventilatory response (HVR), is considered a measure of peripheral chemoreflex sensitivity (liters per minute per % oxygen saturation). **(C,D)** show the effect of isocapnic CO_2_ tension (PCO_2_) on HVR. Note that the HVR varied with isocapnic PCO_2_ tension; with greater levels of isocapnia, the sensitivity of the peripheral chemoreceptors to hypoxia is increased, resulting in a greater HVR. In contrast, during the poikilocapnic test, PCO_2_ fell as V̇_E_ rose, and the ventilatory response to hypoxia was nearly eliminated. See text for details.

Figure 1 provides an example of the breath-by-breath responses to the transient HVR test with P_ET_CO_2_ maintained at 45 mmHg (Figure 1A) and the resultant HVR (Figure 1B). Figures 1C,D include the HVR data from all four tests. Note that the HVR varied with isocapnic PCO_2_ tension; with greater levels of isocapnia, the sensitivity of the peripheral chemoreceptors to hypoxia is increased, resulting in a greater HVR. In contrast, during the poikilocapnic test, PCO_2_ fell as V̇_E_ rose, and the ventilatory response to hypoxia was nearly eliminated.

## Limitations of the Transient Hypoxic Test for Identifying Peripheral Chemoreflex Hypersensitivity

This experiment demonstrates that, depending on the selected level of isocapnia, the same individual can have a different HVR. Note also that, with higher P_ET_CO_2_, the regression lines are shifted upward, reflecting increasing ventilatory drive from central chemoreceptors. Had the transient hypoxia not been preceded by 4 min of normocapnic isocapnia, a contribution of the central chemoreceptor to V̇_E_ would also be present in the HVR response. These observations question the validity of such an HVR test to label a peripheral chemoreflex as “exaggerated.” In this specific example, depending on which isocapnic PCO_2_ tension is used, the patient’s HVR is either above or below the proposed definition of “exaggerated” peripheral chemoreflex sensitivity, i.e., 0.75 L min^−1^∙%S_a_O_2_^−1^.

Augmented ventilatory responses to isocapnic and poikilocapnic hypoxia are reportedly present in 30–60% of HFrEF patients (Narkiewicz et al., 1999b; Ponikowski et al., 1999, 2001; Hering et al., 2007; Giannoni et al., 2008, 2009; Despas et al., 2012). However, these data are derived almost exclusively from transient hypoxic tests. As we and others (Read et al., 1977; Duffin, 2011; Powell, 2012) have illustrated, under conditions of unstandardized PCO_2_ control, these tests cannot characterize peripheral chemoreflex sensitivity nor can they reliably discriminate between patients whose sensitivities differ. Also worth considering are that HFrEF patients often exhibit prolonged lung to carotid body circulatory times (Hall et al., 1996) and Cheyne-Stokes breathing patterns (van de Borne et al., 1996), which can further confound the HVR to transient hypoxia by obscuring the alignment of the independent (S_a_O_2_) and dependent (V̇_E_) variables. Consequently, because the HVR test result is dependent on the isocapnic PCO_2_, and methods to appropriately standardize PCO_2_ control during transient hypoxic tests have not been employed, we question whether the current normative data labeling peripheral chemoreflex sensitivity in HFrEF as “augmented” are appropriate.

## Can PCO_2_ be Standardized Such That the HVR Reflects Peripheral Chemoreflex Sensitivity?

Within an individual, the HVR will depend on three variables: (1) the severity of the hypoxic stimulus (PO_2_); (2) the individual’s responsiveness to PO_2_ at the prevailing PCO_2_ (peripheral chemoreflex sensitivity); and (3) the proximity of the prevailing PCO_2_ to the PCO_2_ of the peripheral chemoreflex VRT. To determine peripheral chemoreflex sensitivity from HVR measurements, it requires (at least) two HVR tests at two isocapnic PCO_2_ tensions that are both above the PCO_2_ of the peripheral chemoreflex VRT. In this way, the change in HVR for the change in PCO_2_ gives a slope reflective of the peripheral chemoreflex sensitivity. For example, in Figure 1, it is evident that the peripheral chemoreflex VRT resides at a PCO_2_ between 39 and 42 mmHg. Therefore, the difference in HVR at 42 and 45 mmHg of PCO_2_ (0.87 vs. 0.71 L·min^−1^·%^−1^) divided by the change in PCO_2_ (45 vs. 42 mmHg) gives 0.05 L·min^−1^·%^−1^ mmHg^−1^, an estimate of peripheral chemoreflex sensitivity, standardized for PCO_2_, that can be appropriately compared to other individuals. Note that, in this case, the two isocapnic HVR measurements are made at PCO_2_ tensions above the known VRT. However, the VRT is unknown in steady-state experiments and cannot be assumed to coincide with eupneic PCO_2_. Moreover, the HVR has to be measured: (i) after the central respiratory chemoreflex response to isocapnia establishes a steady state; and (ii) for a brief duration to minimize the impact of hypoxic ventilatory decline on HVR. Importantly, in HFrEF, methods to appropriately standardize PCO_2_ during transient hypoxic tests have not been employed.

## An Alternative Method to Assess Peripheral Chemoreflex Hypersensitivity in HFrEF

To circumvent the issues associated with standardizing the effect of PCO_2_ on the peripheral chemoreflex response to hypoxia (i.e., HVR), we suggest the reverse, measuring the effect of hypoxia on the peripheral chemoreflex response to PCO_2_. This measurement recognizes that the carotid chemoreceptors are [H^+^] sensors with excitability to CO_2_ that is modulated by PO_2_ (Torrance, 1996); Duffin introduced a rebreathing protocol specifically designed to characterize the responsiveness of the peripheral chemoreflex in humans (Duffin, 2007). The ventilatory response to graded hypercapnia is measured twice, with end-tidal PO_2_ maintained throughout rebreathing at high (P_ET_O_2_ > 150 mmHg) or low (P_ET_O_2_ < 70 mmHg) tensions. In some species, central and peripheral respiratory drives have been shown to interact, as demonstrated by several laboratories (Day and Wilson, 2009; Smith et al., 2015), but this is not the case in humans in whom the hypoxic condition summates the contributions of the central and peripheral chemoreceptors to the net ventilatory drive (Clement et al., 1992, 1995; St Croix et al., 1996; Cui et al., 2012). Consequently, the difference between the hyperoxic and hypoxic test results yields the peripheral chemoreflex responsiveness (Duffin, 2007). Evidence of this physiological principle is presented in detail in the Supplementary Material.

The test is preceded by 3–5 min of volitional hyperventilation to reduce CO_2_ stores and initiate rebreathing from a PCO_2_ below the threshold PCO_2_ at which the chemoreceptors initiate an increase in ventilation (i.e., VRT). The inspiratory bag contains a PCO_2_ close to resting venous during hyperventilation (~35 mmHg) such that initiation of rebreathing, post-hyperventilation, causes rapid equilibration of inspired, alveolar, arterial, and venous PCO_2_ and temporal alignment of the stimulus (i.e., CO_2_) for both central and peripheral chemoreceptors (Read, 1967; Duffin, 2011). Throughout rebreathing, inspired CO_2_ is a function of the previously expired PCO_2_ creating a “ramp” function of PCO_2_ – the slope of the ventilatory response gives the sensitivity in L∙min^−1^∙mmHg^−1^. Importantly, the preceding hyperventilation period, which does not induce short-term potentiation of breathing (Rapanos and Duffin, 1997), permits identification of the VRT, and the rebreathing ramp duration (~4 min) is short enough to avoid hypoxic ventilatory decline (Duffin, 2007). The interested reader seeking a more detailed exposition of the body of literature validating the assumptions intrinsic to this rebreathing protocol is invited to read the accompanying Supplementary Material, as well as previous work by Duffin and others (Duffin, 2007, 2011; Ainslie and Duffin, 2009; Powell, 2012; Duffin and Mateika, 2013; Guyenet et al., 2018).

**Figure 2:**
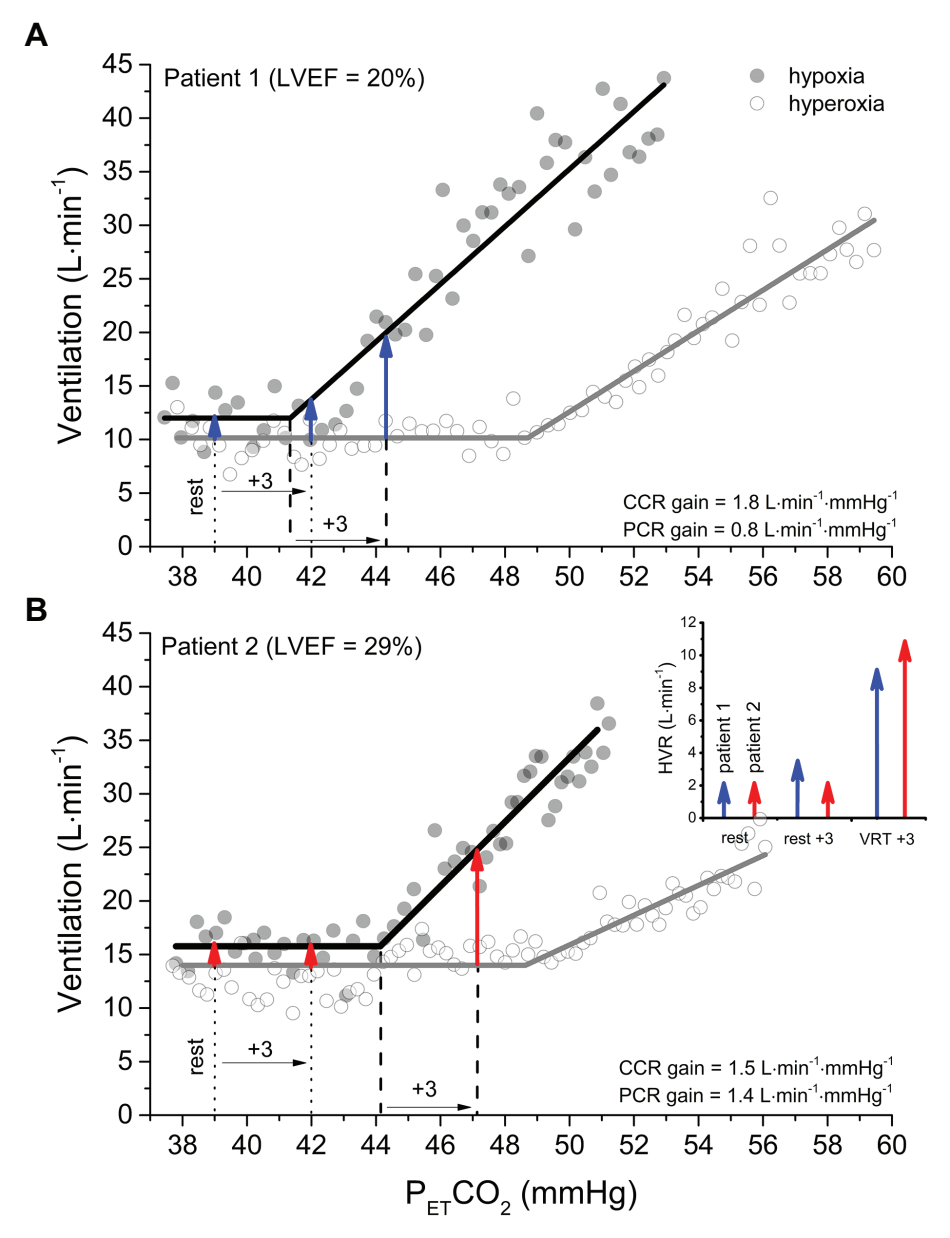
A comparison of isoxic hyperoxic (PO_2_ = 150 mmHg) ventilatory responses to CO_2_ (*white* circles and *gray* lines) with isoxic hypoxic (PO_2_ = 50 mmHg) ventilatory responses to CO_2_ (*black* circles and *black* lines) between two patients with heart failure with reduced ejection fraction. Note that the gain (or sensitivity) is less in patient 1 (A) vs patient 2 (B). However, the ventilatory recruitment threshold (VRT) for the peripheral chemoreflex (PCR) occurs at a higher PCO_2_ in patient 2 vs. 1. The arrows provide an estimation of the HVR attributable to the PCR at isocapnic PCO_2_ corresponding to rest, +3 mmHg above rest, and + 3 mmHg above the VRT for patient 1 (*blue* arrows) and patient 2 (*red* arrows). After determining the ventilation at an isocapnic PCO_2_ in hyperoxia to measure the central chemoreflex (CCR) contribution, hypoxia is introduced, and the ventilation measured again at the isocapnic PCO_2_. Because patient 2 has a great VRT than patient 1, the HVR corresponding isocapnic PCO_2_ at rest and + 3 mmHg above rest incorrectly indicate that the PCR sensitivity of patient 1 is the same or greater than patient 2. By contrast, when hypoxia is introduced at an isocapnic PCO_2_ of +3 mmHg above the VRT, the HVR correctly reveals the PCR gain of patient 2 is greater than patient 1.

Figure 2A shows the ventilatory response to hyperoxic (P_ET_O_2_ = 150 mmHg; O_2_sat = 100%) and hypoxic (P_ET_O_2_ = 50 mmHg; O_2_sat = 83%) rebreathing in the same patient as depicted in Figure 1. Because a P_ET_O_2_ of ~150 mmHg largely desensitizes the peripheral chemoreflex (Lloyd et al., 1957; Lahiri et al., 1993) without independently stimulating ventilation (Becker et al., 1996; also see Supplementary Material), the PCO_2_ at which V̇_E_ begins to rise gives the VRT of the central chemoreflex (i.e., 49 mmHg) and the slope of the linear rise in V̇_E_, thereafter giving its sensitivity or gain (i.e., 1.8 L min^−1^ mmHg^−1^). The gain of the V̇_E_ vs. P_ET_CO_2_ response in the hypoxic trial reflects the additive effects of simultaneous central and peripheral chemoreceptor stimulation (i.e., 2.6 L∙min^−1^ mmHg^−1^) and, thus, the difference in gain between hypoxic and hyperoxic trials yields the peripheral chemoreflex sensitivity (0.8 L∙min^−1^∙mmHg^−1^).

Under hypoxic conditions, the VRT of the peripheral chemoreflex is revealed (i.e., 41 mmHg). Equally important is that the PCO_2_ at the VRT from modified rebreathing does not translate equally to steady-state experiments due to the reinstitution of a central-arterial PCO_2_ difference, which, depending on its magnitude, can contribute to a 2–4 mmHg reduction in VRT (Mohan et al., 1999; Ainslie and Duffin, 2009). Rebreathing vs. steady-state differences notwithstanding, this feature of the peripheral chemoreflex is of critical importance in the evaluation of its sensitivity, because it dictates that HVR measured from an acute hypoxic stimulus depends on PCO_2_. For example, the resting P_ET_CO_2_ of the patient-participant featured in Figure 1A is 39 mmHg, which is ~2 mmHg, i.e., below the VRT of the peripheral chemoreflex. Assuming that the difference between hypoxic and hyperoxic responses provides an estimate of the acute HVR at any given PCO_2_, if the patient was exposed to a bolus of hypoxic gas (e.g., P_ET_O_2_ = 50 mmHg; O_2_sat = 83%) with P_ET_CO_2_ isocapnic at 39 mmHg, one would expect very little change in V̇_E_ (HVR < 2 L min^−1^; see the first blue arrow in Figure 2A). By contrast, the same hypoxic stimulus applied to this patient with P_ET_CO_2_ isocapnic at 44 mmHg (+3 mmHg above VRT) would elicit an HVR that is 5-fold greater (~10 L min^−1^). These important aspects of the peripheral chemoreflex are internally consistent with the data presented in Figure 1D: the HVR of the patient is quite low with P_ET_CO_2_ maintained below the VRT (i.e., 39 mmHg) but increases dramatically with P_ET_CO_2_ maintained at +1 (42 mmHg) and + 4 mmHg (45 mmHg) above the peripheral chemoreflex VRT.

The blue arrows signifying HVR in Figure 2A assume for simplicity that the PCO_2_ at the peripheral and central chemoreceptors is identical. However, it is important to note that a central-arterial PCO_2_ difference of ~10 mmHg exists, which varies depending on PaCO_2_ *via* its effect on medullary blood flow (Ainslie and Duffin, 2009). For this reason, at any isocapnic P_ET_CO_2_, the magnitude of HVR will reflect peripheral chemoreceptor sensitivity only after central chemoreceptor responses to the altered medullary PCO_2_ attain a steady state. Figure 1C demonstrates this effect: with higher isocapnic PCO_2_ tensions, the regression lines are shifted increasingly upward (even at normal arterial O_2_ saturation) due to heightened ventilatory drive from the central chemoreceptors. Such confounding factors must be considered when evaluating HVR.

Consideration of the VRT of the peripheral chemoreflex becomes even more important when the objective is to compare HVR between patients with a condition such as HFrEF, whether for prognostic or interventional purposes. Figure 2B displays the ventilatory response to a modified rebreathing test for a 43-year-old female with dilated cardiomyopathy (NYHA class II, LVEF = 29%; BMI = 37 kg∙m^−2^). Compared to the patient in Figure 2A, her resting P_ET_CO_2_ is the same (39 mmHg), but both her peripheral chemoreflex sensitivity and VRT are higher (1.4 vs. 0.8 L∙min^−1^∙mmHg^−1^ and 44 vs. 41 mmHg, Patient 2 vs. Patient 1, respectively). The graph inset within Figure 2B illustrates the predicted HVR for both patients with P_ET_CO_2_ maintained at rest, +3 mmHg above rest, and + 3 mmHg above VRT. Note that only when measured at a standard P_ET_CO_2_ increment above each patients’ VRT will the HVR correctly indicate that the peripheral chemoreflex sensitivity of Patient 2 is greater than Patient 1. For these reasons, transient hypoxic HVR tests are only capable of identifying differences in peripheral chemoreflex sensitivity between patients when performed at the same P_ET_CO_2_ increment above the peripheral chemoreflex VRT and after central chemoreceptor responses have been given time to stabilize.

## Can the Ventilatory Peripheral Chemoreflex Response in HFrEF be Applied Clinically as a Surrogate for the Sympathetic Response?

Prior research has focused on ventilation as the primary efferent arm of the peripheral chemoreflex and few studies have examined both ventilatory and sympathetic responses to hypoxia in HFrEF. Recording simultaneously breath-by-breath V̇_E_ and MSNA from the fibular nerve during modified rebreathing in healthy young men, we published the first in-human characterization of the sympathetic stimulus-response properties of central and peripheral chemoreflexes (Keir et al., 2019). In response to graded hypercapnia, we discovered that, like ventilation below a threshold PCO_2_, MSNA is unchanged in both hypoxic and hyperoxic conditions, while above this PCO_2_ threshold, MSNA rises linearly with PCO_2_.

The linear rises in MSNA and ventilation occurred simultaneously above similar PCO_2_ thresholds suggesting, in accordance with previous thinking (Guyenet, 2014), that chemoreflex-mediated ventilatory and sympathetic responses to peripheral chemoreceptor stimulation are initiated by common afferent input to the central nervous system. However, when the rates of rise in either V̇_E_ and MSNA were plotted against changes in P_ET_CO_2_, there was no correlation between such slope or sensitivities. Consistent with the notion that the carotid body acts *via* distinct pathways in the regulation of sympathetic and respiratory output (Zera et al., 2019), these findings challenge current beliefs that those who breathe vigorously with peripheral chemoreceptor activation incur an equally vigorous noradrenergic response (i.e., their sensitivities are not equivalent). Moreover, amplified hemodynamic swings plus stimulation of pulmonary stretch receptors, consequent to the ventilatory instability engendered by a hypersensitive chemoreflex, will perturb MSNA indirectly, *via* baroreceptor- and ancillary-reflex mechanisms. Inter-individual differences in carotid chemoreceptor-baroreceptor interactions may be one source of such variability in health and disease (Somers et al., 1991; Janssen et al., 2018; Heusser et al., 2020). Although derived from experiments involving healthy young men, without contrary data from human HFrEF patients, such findings caution against therapeutic targeting of the carotid body solely on the basis of ventilatory responsiveness to hypoxia.

Carotid body tonicity, inferred by the magnitude of the fall in either ventilation or MSNA in response to transient hyperoxia at eupneic PCO_2_, could also identify HFrEF patients who might benefit from carotid body intervention. However, to our knowledge, no longitudinal studies thus far have associated peripheral chemoreceptor tone to survival in this population. Importantly, a recent publication, involving cohorts with hypertension and obstructive sleep apnea, subjected to steady-state hypoxia with PCO_2_ maintained eucapnic also documented discordance between reflex ventilatory and sympathoneural response to this stimulus (Prasad et al., 2020).

## Conclusion

Several recent original contributions and reviews identify the peripheral chemoreceptors as a source of sympathetic excitation in HFrEF and as a therapeutic target (Niewinski et al., 2013, 2017; Paton et al., 2013; Schultz et al., 2013; Marcus et al., 2014b; Del Rio et al., 2015; Niewinski, 2017; Toledo et al., 2017). But, are present data and testing methods of peripheral chemoreflex sensitivity sufficient to justify interventions as radical as carotid body resection or denervation? This focus on the carotid body as a therapeutic target is largely based on a reportedly high prevalence and independent prognostic value of augmented peripheral chemoreflex HVR (i.e., sensitivity) in the HFrEF population measured from brief hypoxic exposures (Chua et al., 1997; Ponikowski et al., 2001; Giannoni et al., 2009) rather than the sympathetic responsiveness *per se*. Importantly, this method of assessing peripheral chemoreflex sensitivity has known intrinsic limitations (Duffin, 2007, 2011; Powell, 2012), and the assumption of concordance between the ventilatory and sympathetic arms of the peripheral chemoreflex in HFrEF has been refuted in healthy individuals (Keir et al., 2019) and in those with OSA (Prasad et al., 2020). Investigations in HFrEF are underway (Keir et al., 2020). Before patients are recommended for well-intentioned interventions, such as carotid body ablation, on the basis of the current transient HVR test (Niewinski et al., 2013; Niewinski, 2017), it would be prudent first to establish the optimum testing methodology and to generate and validate normative and pathological test values. The advantages of the rebreathing test that we propose are that it addresses the critical importance of identifying, for each individual studies, the personal threshold at which PCO_2_ triggers the ventilatory and sympathetic peripheral chemoreflexes when endeavoring to calculate her or his peripheral chemoreflex sensitivity slope; it rectifies the deficiencies of other previously published methods, and having been adopted without difficulty by laboratories without prior expertise, it is demonstrably feasible in practice.

## Data Availability Statement

The raw data supporting the conclusions of this article will be made available by the authors, without undue reservation.

## Ethics Statement

The studies involving human participants were reviewed and approved by University Health Network Research Ethics Board. The patients/participants provided their written informed consent to participate in this study.

## Author Contributions

All experiments were performed in the Clinical Cardiovascular Physiology Laboratory (6ES413), Department of Medicine, Toronto General Hospital. DK, JD, and JF contributed to the conception and design of the experiments. DK collected, analyzed, and interpreted data and drafted and revised the manuscript. JD and JF contributed to analysis and interpretation of data, and drafting and revising of the manuscript. All authors approved the manuscript and agree to be accountable for all aspects of the work.

### Conflict of Interest

JD has equity in Thornhill Medical and receives salary support from Thornhill Medical. Thornhill Medical provided no other support for the study.

The remaining authors declare that the research was conducted in the absence of any commercial or financial relationships that could be construed as a potential conflict of interest.
