# Supplementary material for: Measuring Peripheral Chemoreflex Hypersensitivity in Heart Failure
Source: Front Physiol. 2020 Dec 29;11:595486. doi: 10.3389/fphys.2020.595486 (PMC7802759; doi:10.3389/fphys.2020.595486)
Supplement: Supplementary file 1 [file Presentation_1.pdf]

Measuring peripheral chemoreflex hypersensitivity in heart failure: Supplement

Daniel A. Keir, James Duffin, and John S. Floras

In our Perspectives essay, we used specific examples to demonstrate how a modified rebreathing test surmounts the deficiencies of conventional methods of estimating peripheral chemoreflex sensitivity in human heart failure and can identify the sensitivities and recruitment thresholds of both the ventilatory and sympathoneural efferent responses to peripheral chemoreflex perturbation. In this Supplement, we summarize the physiological principles and observations underlying the modified rebreathing test and provide more detailed responses to the thoughtful questions raised by our knowledgeable referees than can be accommodated within a conventional Perspectives format.

Seminal experiments in the 1950s demonstrated that, in resting humans, CO_2 determines the ventilatory response to acute hypoxia (Nielsen and Smith, 1952) and that "this powerful CO_2 effect is only apparent above a certain value of the alveolar PCO_2 which is close to the threshold value of CO_2 in normal air" (Asmussen and Nielsen, 1956). Within an individual, the ventilatory response to acute hypoxia (HVR) will depend on three variables: 1) the severity of the hypoxic stimulus (PO_2); 2) the individual's responsiveness to PO_2 at the prevailing PCO_2 (peripheral chemoreflex hypoxia sensitivity); 3) the proximity of the prevailing PCO_2 to the PCO_2 associated with the peripheral chemoreflex ventilatory recruitment threshold (VRT).

Contemporary steady-state methods attempt to expose peripheral chemoreflex sensitivity (variable 2) by applying a low PO_2 stimulus with PCO_2 held eucapnic or at a fixed increase in PCO_2 above eucapnia. As shown in our Perspectives, such methods, for two PCO_2-specific reasons, cannot quantify peripheral chemoreflex sensitivity definitively or identifying reliably

inter-individual differences in peripheral chemoreflex gain. The first of these reasons is that the proximity of PCO_2 to the PCO_2 at the peripheral chemoreflex VRT (variable 3) is not standardized. It is often assumed that resting $P_{ET}CO_2$ is equivalent to the PCO_2 at the VRT for the peripheral chemoreflex, but this is not always the case (Mahamed et al., 2001). Thus, it is impossible to know whether the magnitude of the measured HVR reflects peripheral chemoreflex sensitivity (variable 2) or a combination of this and the amount by which isocapnic PCO_2 exceed the VRT (variable 3). Second, because the HVR varies with PCO_2 above the VRT, at least two HVR tests at different levels of isocapnia (both above the PCO_2 associated with the VRT) are needed to ascertain peripheral respiratory chemoreflex sensitivity – one cannot measure sensitivity without constructing a slope! By applying two such tests, the change in HVR for the change in PCO_2 provides a slope reflective of peripheral chemoreflex sensitivity (variable 2) while controlling for variables 1 and 3. But why measure the effect of PCO_2 on the ventilatory response to PO_2 with multiple tests and with unknown VRT when one can measure the effect of PO_2 on the ventilatory response to PCO_2 in a single protocol?

The modified rebreathing protocol does just that: the ventilatory response to the progressive rise in PCO_2 via rebreathing is measured in standardized iso-oxic low and high PO_2 conditions. The high PO_2 condition provides the central chemoreflex response to PCO_2 and the low PO_2 condition gives the response of both central and peripheral respiratory chemoreflexes. The difference in response slopes above the VRT between conditions provides the peripheral chemoreflex sensitivity to CO_2. Importantly, this measure of peripheral chemoreflex sensitivity is derived under standardized PO_2 conditions and is not affected by between-individual differences in VRTs. Initiating rebreathing from a hypocapnic state after a period of slow deep breathing also permits identification of the VRTs for the central (hyperoxic condition) and peripheral (hypoxic

condition) chemoreflexes (although caution should be taken when translating the PCO_2 at the VRT from rebreathing to steady-state experiments (Mohan et al., 1999)).

The modified rebreathing test, therefore, characterizes the CO_2 stimulus-response characteristics of both central and peripheral respiratory chemoreflexes and has the distinct advantage of providing a means by which they can be compared between individuals and within individuals (Jensen et al., 2010) before and after specific interventions (Fan et al., 2010). Since the rebreathing responses describe the central and peripheral chemoreflex characteristics, these can be incorporated into models of the control of breathing (Mahamed et al., 2001; Duffin, 2010).

Several assumptions underly the modified breathing protocol and the interpretation of the measured ventilatory responses. The subsequent section will list each assumption and, briefly, present evidence for their validity. In addition, common critiques of the protocol will be addressed.

1. Assumptions

1.1 Hyperoxia silences the peripheral chemoreflex response to CO_2

The relationship between the ventilatory response to PCO_2 and PaO_2 is well described by a rectangular hyperbola with minimal rise until PaO_2 falls below ~85 mmHg (Lloyd et al., 1957; Duffin et al., 2000). As isoxic PO_2 increases much above 150 mmHg, further reduction in the CO_2 ventilatory response slope becomes insignificant (Lloyd et al., 1957; Mohan and Duffin, 1997; Duffin et al., 2000). Both *in vitro* and *in vivo* experiments demonstrate that a PO_2 of 150 mmHg nearly abolishes Type I cell excitation (Fitzgerald and Parks, 1971; Duchen and Biscoe, 1992; Buckler and Vaughan-jones, 1994; Dasso et al., 2000) and carotid sinus afferent discharge (Hornbein and Roos, 1963; Lahiri and Delaney, 1975; Lahiri et al., 1993; Vidruk et al., 2001). It is possible that, in humans, afferent output from the carotid bodies may still exist in hyperoxia (albeit with a discharge frequency that is largely diminished), but importantly, this afferent input

seems insufficient to generate a reflex response to CO_2. Thus, in our experience, the ventilatory response to PCO_2 during hyperoxic rebreathing reflects reflex responses initiated by the central chemoreceptors.

1.2 Ventilation is controlled by both central and peripheral chemoreflexes and their drives are additive

Anesthetized animal preparations that isolate and control perfusate at the carotid body and medulla suggest that the central and peripheral respiratory chemoreflexes interact hypo-additively in rats (Day and Wilson, 2007) and hyper-additively in canines (Blain et al., 2010; Smith et al., 2015), respectively. In humans the central and peripheral drives to breathe are additive.

In a conscious resting human, there is a ~10 mmHg difference between arterial (~40 mmHg) and brain (~50 mmHg) PCO_2. Although brain PCO_2 is, in part, determined by arterial PCO_2, differences in local metabolism and blood flow can cause the arterial-brain PCO_2 difference to widen or narrow dynamically in response to a given steady-state CO_2 stimulus (note that these dynamics are irrelevant in modified rebreathing where the arterial-venous PCO_2 difference is minimized and PCO_2 rises similarly at the carotid body and medulla; see below). In humans, the speed of ventilatory responsiveness to step-changes in PO_2 and PCO_2 has been used to test central and peripheral chemoreflex interactions. In response to a step-increase in $P_{ET}CO_2$, arterial PCO_2 increases instantaneously whereas central PCO_2 takes longer to adjust due to slow central tissue compartment kinetics (Farhi and Rahn, 1960). Therefore, the rapid response to a step-decrement in PO_2 or increment in PCO_2 is thought to be mediated almost entirely by the peripheral chemoreceptors.

Adopting this approach, the bulk of human evidence suggests that the central and peripheral chemoreflexes do not interact and their inputs are additive. For example, Cui et al., (2012) showed

that the magnitude of the ventilatory response to a step-decrement in PO_2 was the same in the presence of low (prior hyperventilation) and high (prior isocapnic hypercapnia) central PCO_2. Using a similar temporal separation technique, other studies have failed to provide evidence that peripheral chemoreflex ventilatory responses are modulated by central chemoreceptor stimulation (Clement et al., 1992, 1995; St Croix et al., 1996).

Most importantly, isoxic hypoxic rebreathing exposes both central and peripheral chemoreflexes to the same ‘PCO_2 ramp’ stimulus. In response to such simultaneous stimulation, the net ventilatory response (reflecting contributions from both reflex arcs) is consistently linear (Duffin et al., 2000; Preston et al., 2008; Jensen et al., 2010; Keir et al., 2019), and incompatible with a hypo- or hyper-additive response (Duffin and Mateika, 2013)

Two other studies are often cited as evidence for a central-peripheral respiratory chemoreflex interaction in humans:

Dahan et al., (2007) reported that the “slow” ventilatory response to multifrequency binary sequence changes in $P_{ET}CO_2$ (with gain attributable to the central chemoreflex) was reduced after the “fast” ventilatory response (with gain attributable to the peripheral chemoreflex) was abolished by bilateral carotid body resection in three individuals. However, a major limitation of the study is the use of end-tidal forcing to control central PCO_2. With end-tidal forcing the central PCO_2 is amenable to any cerebrovascular and cardiovascular changes that might alter the arterial-brain PCO_2 gradient. Unfortunately, the authors did not track changes in cardiovascular or cerebrovascular dynamics and therefore, it is impossible to tell whether temporal reductions in the magnitude of the ventilatory response to the same step increment in $P_{ET}CO_2$ was related to absence of a hyper-additive input from the peripheral chemoreceptors or because their absence facilitated

greater cerebral blood flow changes (perhaps by a reduction in sympathetic outflow) with step-increments in $P_{ET}CO_2$ such that central PCO_2 increased to a lesser extent.

Teppema et al., (2010) observed that the HVR at equal arterial hydrogren ion concentration ($[H^+]$) increased with higher arterial PCO_2 and, thus, central PCO_2. However, we disagree that this provides evidence of a hyperadditive central-peripheral chemoreflex interaction. It is often assumed that arterial $[H^+]$ is the stimulus for type I glomus cell excitation. However, it is the intracellular $[H^+]$ that causes excitation. The factors that can be manipulated *in vivo* to determine intracellular $[H^+]$ are $PaCO_2$ and arterial $[H^+]$. Even if arterial $[H^+]$ is held constant, a rise in $PaCO_2$ will still cause an increase in intracellular $[H^+]$ and carotid chemoreceptor cell depolarization. Therefore, in an intact human it is impossible to isolate the effects of $PaCO_2$ and arterial $[H^+]$ on the "sensed" stimulus, i.e., intracellular $[H^+]$. The increase in HVR observed by Teppema et al., (2010) at constant arterial $[H^+]$, likely resulted from a $PaCO_2$-induced rise in intracellular $[H^+]$ at the carotid body and does not support a hyper-additive effect of central chemoreceptors.

1.3 Arterial and medullary chemoreceptor CO_2 are equal during rebreathing

Experimental testing of this assumption is difficult to obtain in humans. The initial PCO_2 in the rebreathing bag is approximately equal to that in venous circulation (~35 mmHg). The transition from hyperventilation to rebreathing simply stops excretion of CO_2 at the lungs so that PCO_2 in the arterial and venous circulation rise in unison at a rate determined by metabolic production of CO_2 and body CO_2 stores. Mathematical simulations based on compartment models (for example the original test model by Read and Leigh, (1967)) showed that, after the initial equilibration to mixed venous values, the PCO_2 in all compartments rise together, mixed by the circulation. Any PCO_2 difference between compartments is therefore due to circulatory time delays. Assuming a circulatory time delay of ~10 s from the central compartment to the lung

compartment and a rate of rise of CO_2 during rebreathing of about 0.07 mmHg/s, a difference of 0.7 mmHg may be expected but this would be reduced as CO_2 increases cerebral blood flow.

2. Critiques

2.1 Prior voluntary hyperventilation produces short-term potentiation that will alter ventilatory responses to CO_2 independent of the chemoreflexes

It is important to note that the hyperventilation prior to rebreathing is of a slow, deep pattern akin to that used in meditation and therefore is more likely to induce relaxation rather than excitation. The effect of short-term potentiation has been studied. Chatha and Duffin, (1997) found no short-term potentiation during rebreathing and Mohan et al. (1999) demonstrated that the ventilatory response to hyperoxic rebreathing was the same with and without prior hyperventilation. Other studies support that most healthy individuals do not display hyperpnea after ~12 s post-hyperventilation (Mahamed et al., 2004) and even apnea has been reported for some subjects rather than short-term potentiation (Skatrud and Dempsey, 1983; Meah and Gardner, 1994; Mateika and Ellythy, 2003). In most individuals performing modified rebreathing, the onset of the linear rise in ventilation occurs ~1.5 minutes after rebreathing onset in hypoxia (the PCO_2 threshold is further delayed in hyperoxia). Any hyperpnea, if present, is not included in the model parameter estimates either by excluding these data or by fitting the data below VRT an exponential decay model (e.g. (Jensen et al., 2010)). Therefore, even if a brief period of hyperpnea is present, it is unlikely to influence the baseline, VRT or slope parameters describing the respiratory chemoreflexes. Furthermore, to our knowledge, there is no evidence that short-term potentiation has any effect other than raising basal ventilation and thus, if present, it is unlikely that the PCO_2 VRT or ventilatory responsiveness to PCO_2 are affected.

2.2 Hyperoxia will stimulate ventilation independent of its effect on CO_2 responsiveness

This statement suggests that the ventilatory response to iso-oxic hyperoxic rebreathing will reflect the central respiratory chemoreflex response plus a non-chemoreflex mediated response to high PO_2. The findings of Becker et al., (1996) often are cited to support this assertion. With respect to the Becker data, it should be emphasized that the increase in ventilation with 30% O_2 breathing likely was related to central chemoreceptor stimulation by PCO_2. Those experiments were performed in steady-state isocapnic conditions, where central PCO_2 would be expected to rise progressively (relative to arterial PCO_2) with hyperoxia due to the Haldane effect (Eldridge and Kiley, 1987). When their experiment was repeated under poikilocapnic conditions the hyperoxic ventilatory response disappeared.

Unlike steady-state experiments, rebreathing uncouples hyperoxia from its affects on central PCO_2 and thereby the central chemoreflex drive to ventilation is isolated. Arterial and medullary PCO_2 are equivalent and rise progressively and synchronously with time (Read and Leigh, 1967). Finally, modified rebreathing utilizes a very mild PO_2 at which to maintain iso-oxic $P_{ET}O_2$ (~150 mmHg or ~26% FiO_2).

2.3 $PaCO_2$ does not accurately reflect the central chemoreceptor PCO_2 across subjects who differ in their cerebral blood flow sensitivities to PO_2 and/or PCO_2

Individual-specific differences in cerebral blood flow sensitivities to PO_2 and PCO_2 are only relevant in steady-state experiments and are irrelevant during rebreathing. During rebreathing, arterial-venous PCO_2 differences are minimal. Any rise in cerebral blood flow with CO_2 (or hypoxia) will contribute to a further reduction in the arterial to central PCO_2 difference.

2.4 Hypoxic ventilatory decline will affect the ventilatory response to PCO_2 in hypoxia

The isocapnic hypoxic ventilatory response in steady-state conditions can arbitrarily divided into two phases with an immediate increase (0-5 minutes) followed by a slow "hypoxic ventilatory decline (HVD)" (5-20 minutes) (Steinback and Poulin, 2007). While HVD is an important consideration in steady-state experiments, it is not significant in modified rebreathing where isoxic hypoxic tests are completed in less than 4 minutes.

3. Summary

In a normal resting human, there is an ~10 mmHg difference between arterial (~40 mmHg) and brain (~50 mmHg) PCO_2. Although brain PCO_2 is, in part, determined by arterial PCO_2 (and PO_2) differences in local metabolism and blood flow can cause the arterial-brain PCO_2 difference to widen or narrow dynamically in response to a given CO_2 stimulus. By minimizing (or eliminating) this difference, modified rebreathing is the only method that is capable of separating central from peripheral respiratory chemoreflexes in humans to confidently quantify their independent reflex responsiveness and, from the clinical perspective, identify with greater precision those heart failure patients most likely to benefit from carotid body interventions.

References

Asmussen, E., and Nielsen, M. (1956). Ventilatory Response to CO2 During Work at Normal and at Low Oxygen Tensions. *Acta Physiol. Scand.* 39, 27–35. doi:10.1111/j.1748-1716.1957.tb01406.x.

Becker, H. F., Polo, O., Mcnamara, S. G., Berthon-Jones, M., and Sullivan, C. E. (1996). Effect of different levels of hyperoxia on breathing in healthy subjects. *J. Appl. Physiol.* 81, 1683–1690.

Blain, G. M., Smith, C. A., Henderson, K. S., and Dempsey, J. A. (2010). Peripheral chemoreceptors determine the respiratory sensitivity of central chemoreceptors to CO_2. *J Physiol* 58813, 2455–2471. doi:10.1113/jphysiol.2010.187211.

Buckler, K. J., and Vaughan-jones, R. D. (1994). Effects of hypoxia on membrane potential and intracellular calcium in rat neonatal carotid body type I cells. *J. Physiol.* 476, 423–428.

Chatha, D., and Duffin, J. (1997). The pattern of breathing following a 10-breath voluntary hyperventilation during hyperoxic rebreathing. *Can. J. Appl. Physiol.* 22, 256–267.

Clement, I. D., Bascom, D. A., Conway, J., Dorrington, K. L., O’Connor, D. F., Painter, R., et al. (1992). An assessment of central-peripheral ventilatory chemoreflex interaction in humans. *Respir. Physiol.* 88, 87–100. doi:10.1016/0034-5687(92)90031-Q.

Clement, I. D., Pandit, J. J., Bascom, D. A., Dorrington, K. L., O’Connor, D. F., and Robbins, P. A. (1995). An assessment of central-peripheral ventilatory chemoreflex interaction using acid and bicarbonate infusions in humans. *J. Physiol.* 485, 561–570. doi:10.1038/nm0898-934.

Cui, Z., Fisher, J. A., and Duffin, J. (2012). Central-peripheral respiratory chemoreflex interaction in humans. *Respir. Physiol. Neurobiol.* 180, 126–131. doi:10.1016/j.resp.2011.11.002.

Dahan, A., Nieuwenhuijs, D., and Teppema, L. (2007). Plasticity of central chemoreceptors: Effect

of bilateral carotid body resection on central CO2 sensitivity. *PLoS Med.* 4, 1195–1204. doi:10.1371/journal.pmed.0040239.

Dasso, L. L. T., Buckler, K. J., and Vaughan-Jones, R. D. (2000). Interactions between hypoxia and hypercapnic acidosis on calcium signaling in carotid body type I cells. *Am. J. Physiol. Cell. Mol. Physiol.* 279, L36–L42. doi:10.1152/ajplung.2000.279.1.l36.

Day, T. A., and Wilson, R. J. A. (2007). Brainstem PCO_2 modulates phrenic responses to specific carotid body hypoxia in an in situ dual perfused rat preparation. *J. Physiol.* 578, 843–857. doi:10.1113/jphysiol.2006.119594.

Duchen, B. Y. M. R., and Biscoe, T. J. (1992). Relative mitochondrial membrane potential and $[Ca^{2+}]_i$ in type I cells isolated from the rabbit carotid body. *Physiology* 450, 33–61.

Duffin, J. (2010). The role of the central chemoreceptors : A modeling perspective. *Respir. Physiol. Neurobiol.* 173, 230–243. doi:10.1016/j.resp.2010.03.010.

Duffin, J., and Mateika, J. H. (2013). CrossTalk opposing view: Peripheral and central chemoreflexes have additive effects on ventilation in humans. *J. Physiol.* 591, 4351–4353. doi:10.1113/jphysiol.2013.256800.

Duffin, J., Mohan, R. M., Vasiliou, P., Stephenson, R., and Mahamed, S. (2000). A model of the chemoreflex control of breathing in humans: Model parameters measurement. *Respir. Physiol.* 120, 13–26. doi:10.1016/S0034-5687(00)00095-5.

Eldridge, F. L., and Kiley, J. P. (1987). Effects of hyperoxia on medullary ECF pH and respiration in chemodenervated cats. *Respir. Physiol.* 70, 37–49. doi:10.1016/S0034-5687(87)80030-0.

Fan, J. L., Burgess, K. R., Basnyat, R., Thomas, K. N., Peebles, K. C., Lucas, S. J. E., et al. (2010). Influence of high altitude on cerebrovascular and ventilatory responsiveness to CO2. *J. Physiol.* 588, 539–549. doi:10.1113/jphysiol.2009.184051.

Farhi, L. E., and Rahn, H. (1960). Dynamics of changes in carbon dioxide stores. *Anesthesiology* 21, 604–614.

Fitzgerald, R. S., and Parks, D. C. (1971). Effect of hypoxia on carotid chemoreceptor response to carbon dioxide in cats. *Respir. Physiol.* 12, 218–229.

Hornbein, T. F., and Roos, A. (1963). Specificity of H ion concentration as a carotid chemoreceptor stimulus. *J. Appl. Physiol.* 18, 580–584.

Jensen, D., Mask, G., and Tschakovsky, M. E. (2010). Variability of the ventilatory response to Duffin's modified hyperoxic and hypoxic rebreathing procedure in healthy awake humans. *Respir. Physiol. Neurobiol.* 170, 185–197. doi:10.1016/j.resp.2009.12.007.

Keir, D. A., Duffin, J., Millar, P. J., and Floras, J. S. (2019). Simultaneous assessment of central and peripheral chemoreflex regulation of muscle sympathetic nerve activity and ventilation in healthy young men. *J. Physiol.* 597, 3281–3296. doi:10.1113/JP277691.

Lahiri, S., and Delaney, R. G. (1975). Stimulus interacation in the responses of carotid body chemoreceptor single afferent fibers. *Respir. Physiol.* 24, 249–266.

Lahiri, S., Rumsey, W. L., Wilson, D. F., and Iturriaga, R. (1993). Contribution of in vivo microvascular PO2 in the cat carotid body chemotransduction. *J. Appl. Physiol.* 75, 1035–1043. doi:10.1152/jappl.1993.75.3.1035.

Lloyd, B., Jukes, M., and Cunningham, D. J. C. (1957). The Relation Between Alveolar Oxygen Pressure and the respiratory response to carbon dioxide in man. *Q. J. Exp. Physiol.* 43, 214–227.

Mahamed, S., Ali, A. F., Ho, D., Wang, B., and Duffin, J. (2001). The contribution of chemoreflex drives to resting breathing in man. *Exp. Physiol.* 86, 109–116. doi:10.1113/eph8602090.

Mahamed, S., Tsai, J., and Duffin, J. (2004). Cardio-respiratory measures following isocapnic

voluntary hyperventilation. *Respir. Physiol. Neurobiol.* 142, 13–25. doi:10.1016/j.resp.2004.05.005.

Mateika, J. H., and Ellythy, M. (2003). Chemoreflex control of ventilation is altered during wakefulness in humans with OSA. *Respir. Physiol. Neurobiol.* 138, 45–57. doi:10.1016/S1569-9048(03)00174-5.

Meah, M. S., and Gardner, W. N. (1994). Post-hyperventilation apnoea in conscious humans. *J. Physiol.* 477, 527–538. doi:10.1113/jphysiol.1994.sp020213.

Mohan, R. M., Amara, C. E., Cunningham, D. A., and Duffin, J. (1999). Measuring central-chemoreflex sensitivity in man: Rebreathing and steady-state methods compared. *Respir. Physiol.* 115, 23–33. doi:10.1016/S0034-5687(99)00003-1.

Mohan, R. M., and Duffin, J. (1997). The effect of hypoxia on the ventilatory response to carbon dioxide in man. *Respir. Physiol.* 108, 101–115.

Nielsen, M., and Smith, H. (1952). Studies on the Regulation of Respiration in Acute Hypoxia. *Acta Physiol. Scand.* 24, 293–313. doi:10.1111/j.1748-1716.1952.tb00847.x.

Preston, M. E., Jensen, D., Janssen, I., and Fisher, J. T. (2008). Effect of menopause on the chemical control of breathing and its relationship with acid-base status. *AJP Regul. Integr. Comp. Physiol.* 296, R722–R727. doi:10.1152/ajpregu.90865.2008.

Read, D., and Leigh, J. (1967). Blood-brain tissue PCO_2 relationships and ventilation during rebreathing. *J. Appl. Physiol.* 23, 53–70.

Skatrud, J. B., and Dempsey, J. A. (1983). Interaction of sleep state and chemical stimuli in sustaining rhythmic ventilation. *J. Appl. Physiol.* 55, 813–822. doi:10.1152/jappl.1983.55.3.813.

Smith, C. A., Blain, G. M., Henderson, K. S., and Dempsey, J. A. (2015). Peripheral

chemoreceptors determine the respiratory sensitivity of central chemoreceptors to CO_2: role of carotid body CO_2 2. *J. Physiol.* 593, 4225–4243. doi:10.1113/JP270114.

St Croix, C. M., Cunningham, D. A., Paterson, A., and Paterson, D. H. (1996). Nature of the interaction between central and peripheral chemoreceptor drives in human subjects. *Can. J. Physiol. Pharmacol.* 74, 640–646. doi:10.1139/y96-049.

Steinback, C. D., and Poulin, M. J. (2007). Ventilatory responses to isocapnic and poikilocapnic hypoxia in humans. *Respir. Physiol. Neurobiol.* 155, 104–113. doi:10.1016/j.resp.2006.05.006.

Teppema, L. J., Van Dorp, E. L. A., and Dahan, A. (2010). Arterial [H+] and the ventilatory response to hypoxia in humans: Influence of acetazolamide-induced metabolic acidosis. *Am. J. Physiol. - Lung Cell. Mol. Physiol.* 298, 89–95. doi:10.1152/ajplung.00255.2009.

Vidruk, E. H., Burt Olson, E., Ling, L., and Mitchell, G. S. (2001). Responses of single-unit carotid body chemoreceptors in adult rats. *J. Physiol.* 531, 165–170. doi:10.1111/j.1469-7793.2001.0165j.x.
